# Transcriptional regulation of adipocyte lipolysis by IRF2BP2

**DOI:** 10.1126/sciadv.ads5963

**Published:** 2025-01-03

**Authors:** Yang Chen, Lin Liu, Ryan Calhoun, Lan Cheng, David Merrick, David J. Steger, Patrick Seale

**Affiliations:** ^1^ Institute for Diabetes, Obesity and Metabolism, Perelman School of Medicine, University of Pennsylvania, Philadelphia, PA, USA.; ^2^ Department of Cell and Developmental Biology, Perelman School of Medicine, University of Pennsylvania, Philadelphia, PA, USA.

## Abstract

Adipocyte lipolysis controls systemic energy levels and metabolic homeostasis. Lipolysis is regulated by posttranslational modifications of key lipolytic enzymes. However, less is known about the transcriptional mechanisms that regulate lipolysis. Here, we identify interferon regulatory factor–2 binding protein 2 (IRF2BP2) as a transcriptional repressor of adipocyte lipolysis. Deletion of *IRF2BP2* in human adipocytes increases lipolysis without affecting glucose uptake, whereas IRF2BP2 overexpression decreases lipolysis. RNA sequencing, and chromatin immunoprecipitation sequencing analyses show that IRF2BP2 represses lipolysis-related genes, including *LIPE*, which encodes hormone sensitive lipase, the rate-limiting enzyme in lipolysis. Adipocyte-selective deletion of *Irf2bp2* in mice increases *Lipe* expression and free fatty acid levels, resulting in adipose tissue inflammation and glucose intolerance. Together, these findings demonstrate that IRF2BP2 restrains adipocyte lipolysis and opens avenues to target lipolysis for the treatment of metabolic disease.

## Teaser

The transcriptional factor IRF2BP2 represses adipocyte lipolysis and decreases inflammation.

## INTRODUCTION

Adipocytes play a central role in regulating systemic energy levels and metabolic health. Adipocytes store energy as triglyceride in lipid droplets and release energy in the form of free fatty acids (FFAs) and glycerol through lipolysis (*1*). Adipocytes display remarkable plasticity and undergo dynamic changes in their metabolic program in response to many physiologic and pathologic stimuli.

Lipolysis is activated under conditions of energy demand, such as during fasting or exercise. Triglycerides are cleaved into diacylglycerol and fatty acids by the enzyme adipose triglyceride lipase (ATGL), followed by the hydrolysis of diacylglycerol into monoacylglycerol and fatty acids through the action of hormone-sensitive lipase (HSL; encoded by the *LIPE* gene). FFAs and glycerol are released into the bloodstream to provide fuel for other organs. Excessive or dysregulated adipocyte lipolysis leads to ectopic fat deposition in liver, muscle, pancreas, and other organs, driving insulin resistance and glucose intolerance (*2*–*4*). Aberrant lipolysis can also lead to hyperlipidemia and cardiometabolic abnormalities (*5*, *6*). Mutations in key lipolysis genes (e.g., *PNPLA2* and *LIPE*) are associated with human metabolic and cardiac dysfunction, such as lipodystrophy, insulin resistance, and cardiac myopathy (*7*, *8*).

Adipocyte lipolysis is activated by the sympathetic nervous system and various hormones, including cortisol, glucagon, and natriuretic peptides (*4*, *9*–*12*). These stimuli lead to the phosphorylation and activation of lipolysis enzymes ATGL and HSL (*13*–*15*). Conversely, insulin acts on adipocytes to restrain lipolysis (*11*, *16*, *17*). The function of ATGL and HSL are also regulated by other pathways such as cyclic adenosine monophosphate activators, fibroblast growth factor–1, serotonin, and inflammatory factors (*18*–*24*). However, there has been limited research on the transcriptional regulation of lipolysis. Transcriptional mechanisms may be especially important for determining the rate of basal lipolysis. Peroxisome proliferator–activated receptor γ (PPARγ), liver X receptor α, and steroidogenic factor-1 regulate *LIPE* expression in adipocytes (*25*–*27*). Identifying additional transcription factors that regulate lipolysis may provide new therapeutic avenues to ameliorate and/or prevent insulin resistance and related cardiometabolic disorders.

Interferon regulatory factor–2 binding protein 2 (IRF2BP2) is a transcriptional cofactor that governs diverse biological processes including macrophage inflammatory responses, lymphocyte differentiation, cardiomyocyte hypertrophy, and hepatic steatosis (*28*–*31*). IRF2BP2 was first described as an IRF2-dependent transcriptional corepressor but is also reported to activate target genes in certain contexts (*32*, *33*). Notably, *IRF2BP2* variants identified from genome-wide association studies are associated with circulating lipid levels and coronary artery disease risk (*34*). However, the role of IRF2BP2 in regulating adipocyte function was unknown.

In this study, we found that IRF2BP2 represses lipolysis in adipocytes and that this action is required to maintain systemic metabolic homeostasis. In human adipocytes, deletion of *IRF2BP2* elevates lipolysis, whereas overexpression (OE) of IRF2BP2 suppresses lipolysis. Integrated analysis of RNA sequencing (RNA-seq) and chromatin immunoprecipitation sequencing (ChIP-seq) results show that IRF2BP2 binds and represses lipolysis-related genes, including *LIPE*, which encodes the rate-limiting lipolysis enzyme HSL. Adipocyte-selective deletion of *Irf2bp2* in mice increases *Lipe* expression and elevates circulating FFA levels. Consequently, *Irf2bp2* mutant mice exhibit increased adipose tissue and systemic inflammation and glucose intolerance. Together, our results demonstrate that adipocyte IRF2BP2 regulates whole body metabolic homeostasis through transcriptional repression of *LIPE* expression and lipolysis.

## RESULTS

### Loss of *IRF2BP2* increases lipolysis in human adipocytes

**Fig. 1.**
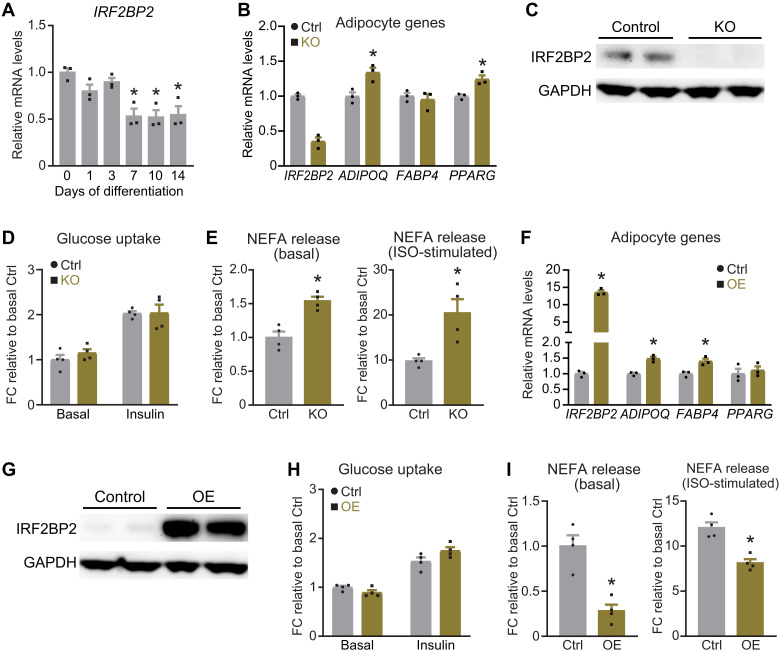
IRF2BP2 regulates adipocyte lipolysis. (**A**) Relative *IRF2BP2* mRNA levels during human adipocyte differentiation (*n* = 3 in each time point). (**B** to **E**) hAPCs were transduced with *IRF2BP2*-targeting (KO) CRISPR lentivirus or nontargeting control lentivirus (Ctrl), and differentiated into adipocytes for 14 days. (B) Relative mRNA levels of *IRF2BP2* and adipocyte marker genes (*ADIPOQ*, *FABP4*, and *PPARG*). (C) Western blot analysis of IRF2BP2 and glyceraldehyde phosphate dehydrogenase (GAPDH) (loading control) protein levels. (D) Glucose uptake in Ctrl and KO adipocytes treated with either phosphate-buffered saline (PBS) or 10^−8^ M insulin. (E) NEFA levels in culture medium from Ctrl and KO adipocytes under basal conditions or following stimulation with 10^−6^ M isoproterenol (ISO). (**F** to **I**) hAPCs were transduced with IRF2BP2-expressing (OE) or GFP-expressing lentivirus (Ctrl) and differentiated into adipocytes for 14 days. (F) Relative mRNA levels of *IRF2BP2*, *ADIPOQ*, *FABP4*, and *PPARG*. (G) Western blot analysis of IRF2BP2 and GAPDH (loading control) protein levels. (H) Glucose uptake in Ctrl and OE adipocytes treated with either PBS or 10^−8^ M insulin. (I) NEFA levels in culture medium from Ctrl and OE adipocytes under basal conditions or following stimulation with 10^−6^ M ISO. Unpaired two-tailed Student’s *t* tests were used in (B), (E), (F), and (I). One-way analysis of variance (ANOVA) followed by Dunnett multiple comparisons test was applied in (A). *P < 0.05.

First, we evaluated the expression of *IRF2BP2* during human adipocyte differentiation. Primary human adipose tissue–derived precursor cells (hAPCs) were induced to differentiate into lipid droplet–containing adipocytes that expressed high levels of adipocyte-selective genes (*ADIPOQ*, *FABP4*, *PPARG*, and *LIPE*) by day 14 (fig. S1, A to C). *IRF2BP2* mRNA levels decreased by ~50% at day 7 and remained lower in mature adipocytes compared to hAPCs (Fig. 1A). Analysis of a single nucleus RNA-seq dataset from human adipose tissue also showed lower *IRF2BP2* mRNA levels in adipocytes relative to hAPCs (fig. S1, D and E) (*35*).

To evaluate the function of IRF2BP2 in human adipocytes, we deleted *IRF2BP2* in hAPCs using a lentiviral CRISPR system and induced adipocyte differentiation for 14 days. *IRF2BP2* mRNA levels were markedly reduced, and protein levels were near absent in *IRF2BP2*-knockout (KO) cells compared to control cells (transduced with nontargeting single-guide RNA) (Fig. 1, B and C). Deletion of *IRF2BP2* had a minimal effect on the adipocyte differentiation process and slightly increased the expression levels of adipocyte marker genes *ADIPOQ* and *PPARG* (Fig. 1B). *IRF2BP2* KO and control adipocytes (at day 14) exhibited equivalent levels of basal and insulin-stimulated glucose uptake (Fig. 1D). *IRF2BP2* KO adipocytes released significantly higher levels of nonesterified fatty acids (NEFAs) and glycerol into the culture medium under basal conditions, as compared to control cells (Fig. 1E and fig. S1F). Isoproterenol treatment greatly increased lipolysis (NEFA and glycerol release) in control and *IRF2BP2* KO adipocytes, with the KO adipocytes attaining ~2-fold higher levels of lipolysis than control cells (Fig. 1E and fig. S1F).

### IRF2BP2 OE in human adipocytes decreases lipolysis

To determine whether IRF2BP2 OE could reduce lipolysis, we transduced hAPCs with control [green fluorescent protein (GFP)] or IRF2BP2-expressing lentivirus and induced adipocyte differentiation for 14 days. IRF2BP2 OE cells expressed high levels of *IRF2BP2* mRNA and protein and slightly increased expression levels of *ADIPOQ* and *FABP4* (Fig. 1, F and G). Control and IRF2BP2-OE adipocytes displayed similar rates of glucose uptake under basal conditions and following insulin stimulation (Fig. 1H). Notably, IRF2BP2 expression decreased lipolysis (NEFA and glycerol release) by ~70% under basal conditions and significantly decreased lipolysis following isoproterenol treatment (Fig. 1I and fig. S1G). Together, these results demonstrate that IRF2BP2 represses lipolysis in human adipocytes without influencing the differentiation process or glucose uptake.

### IRF2BP2 represses lipolysis-related genes in adipocytes

**Fig. 2.**
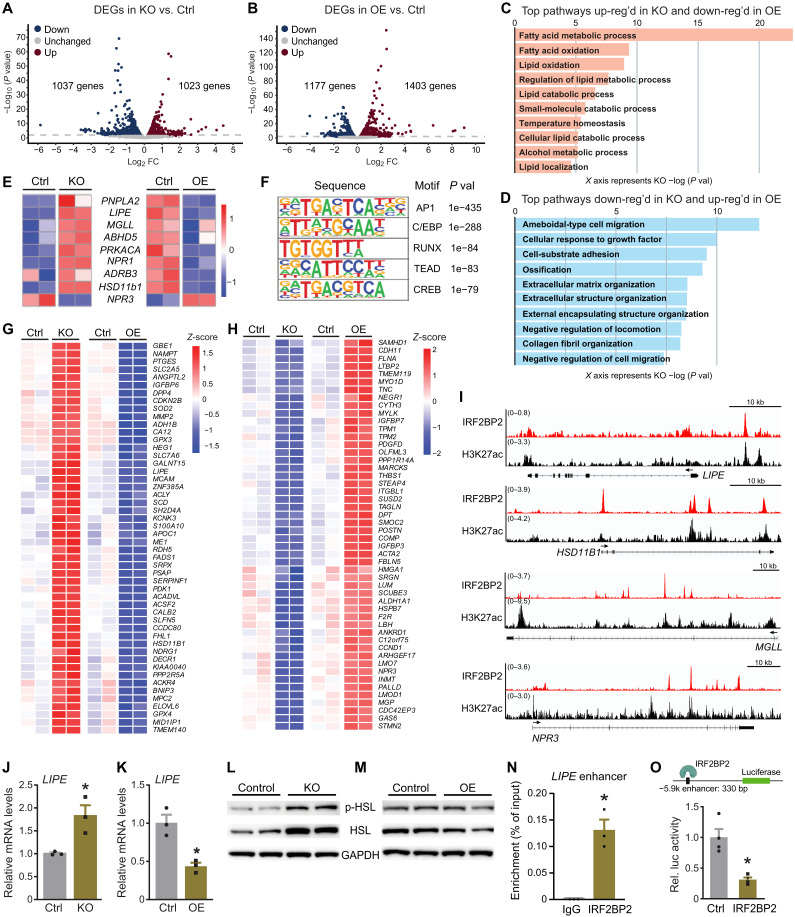
Identification of IRF2BP2 target genes including *LIPE*. (**A** and **B**) Volcano plots showing results from RNA-seq analyses of control (Ctrl) versus *IRF2BP2*-KO (A) and Ctrl versus IRF2BP2-overexpressing (OE) adipocytes (B). *X* axis: Fold changes in mRNA levels (log_2_ transformed) over Ctrl group. *Y* axis: −Log_10_ (*P* value) for significance. (**C** and **D**) Gene enrichment pathway analysis using Biological Processes (BP) database. (C) The top 10 pathways that are (i) up-regulated in KO and (ii) down-regulated in OE adipocytes. (D) The top 10 pathways that are (i) down-regulated in KO and (ii) up-regulated in OE adipocytes. (**E**) Heatmap showing the expression profile of lipolysis genes in KO and OE adipocytes, relative to control cells. (**F**) Motif analysis of IRF2BP2 binding regions in hAPCs subjected to differentiation cocktail for 1 day. (**G** and **H**) Expression heatmap of the top 50 IRF2BP2-repressed (G) and IRF2BP2-activated genes (H) that have nearby IRF2BP2 binding peaks. (**I**) ChIP-seq tracks for IRF2BP2 and H3K27Ac at *LIPE*, *MGLL*, *HSD11B1*, and *NPR3*. (**J** and **K**) Relative *LIPE* mRNA levels in (J) Ctrl and KO adipocytes and (K) Ctrl and OE adipocytes. (**L** and **M**) Western blot analysis of HSL, phospho-HSL, and GAPDH (loading control) levels in (L) Ctrl and KO adipocytes and (M) Ctrl and OE adipocytes. (**N**) ChIP-qPCR analysis for IRF2BP2 or IgG (control) at the −5.9-kb region of *LIPE* in mature hAPC-derived adipocytes. (**O**) Transcription assay showing activity of −5.9-kb region of *LIPE* in immortalized hAPCs transfected with control vector (Ctrl) or IRF2BP2-expressing vector. For (J), (K), (N), and (O), unpaired two-tailed Student’s *t* tests were used. *P < 0.05.

To identify IRF2BP2-regulated genes and pathways in adipocytes, we performed RNA-seq analysis of *IRF2BP2*-KO and *IRF2BP2*-OE adipocytes and their respective control adipocytes (Fig. 2, A and B, and fig. S2, A to F). These studies identified high confidence sets of IRF2BP2-repressed genes (163 genes up-regulated in KO and down-regulated in OE cells) and IRF2BP2-activated genes (175 genes down-regulated in KO and up-regulated in OE cells). Pathway analysis of IRF2BP2-repressed genes identified “fatty acid oxidation” and “lipid catabolic process” as top-ranking terms (Fig. 2C and fig. S2G). Notably, the IRF2BP2-repressed gene set included several classical lipolysis-related genes e.g., *LIPE*, *HSD11B1*, and *PNPLA2* (Fig. 2E). IRF2BP2-activated genes were enriched for pathways related to “cell migration,” “ossification,” and “extracellular matrix organization” (Fig. 2D and fig. S2H).

To identify genes that may be directly regulated by IRF2BP2, we performed ChIP-seq analysis for IRF2BP2 in hAPCs following stimulation with adipogenesis cocktail. Motif analysis of IRF2BP2 binding regions identified a strong enrichment of motifs for several transcription factors, especially activating protein 1 (AP1) and CCAAT/enhancer binding protein (Fig. 2F). Overlapping the RNA-seq and ChIP-seq datasets identified putative direct IRF2BP2 target genes that contained proximal binding sites and displayed IRFBP2-regulated expression (repressed or activated; Fig. 2, G and H). This list included several lipolysis-related genes (i.e., *LIPE*, *MGLL*, *HSD11B1*, and *NPR3*) (Fig. 2, G to I). Many of the IRF2BP2 binding regions displayed peaks of H3K27-acetylation, suggesting that they correspond to regulatory regions (Fig. 2I).

### IRF2BP2 represses *LIPE* transcription

We next focused on the IRF2BP2-regulation of *LIPE*, which encodes the rate-limiting enzyme in adipocyte lipolysis. Quantitative real-time polymerase chain reaction (qRT-PCR) analysis on independent samples showed that *LIPE* mRNA levels were increased (~1.8-fold) by *IRF2BP2* deletion and (~60%) reduced by IRF2BP2 OE in human adipocytes (Fig. 2, J and K). HSL protein levels were similarly regulated, with IRF2BP2-KO cells displaying increased HSL protein levels and IRF2BP2-OE cells displaying lower HSL levels compared to control cells (Fig. 2, L and M). Levels of phosphorylated (activated) HSL followed a similar expression pattern (Fig. 2, L and M).

The ChIP-seq analysis identified a prominent IRF2BP2 binding site 5.9 kb upstream of the *LIPE* transcriptional start site. ChIP–quantitative PCR (qPCR) analyses showed that IRF2BP2 was highly enriched at this −5.9-kb region in mature adipocytes (Fig. 2N). To evaluate whether IRF2BP2 functions at this site, we performed luciferase-based transcription assays in an immortalized hAPC cell line. IRF2BP2 decreased the transcriptional activity of this putative regulatory region by ~70%, relative to the vector control (Fig. 2O). These results suggest that IRF2BP2 represses *LIPE* transcription via binding to the −5.9-kb region.

**Fig. 3.**
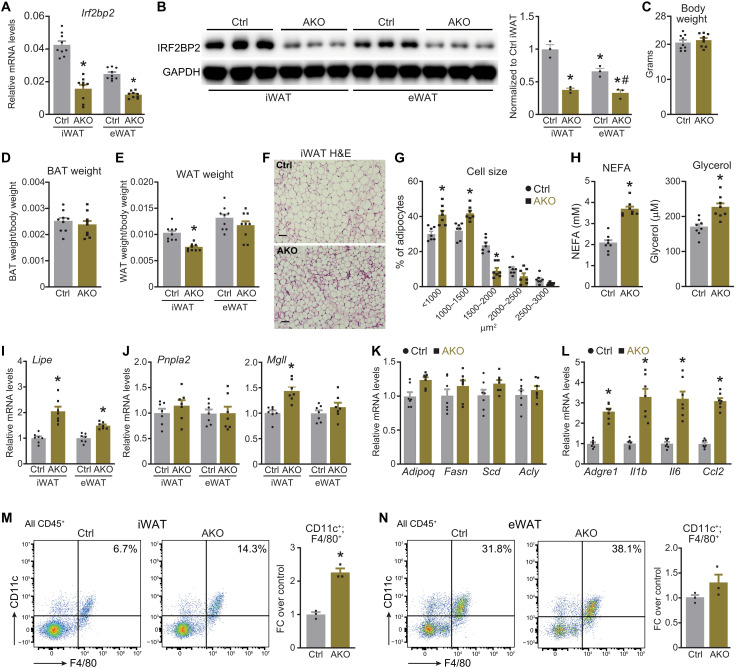
Adipocyte-specific deletion of *Irf2bp2* increases lipolysis and inflammation in adipose tissue. Control and AKO mice were assessed as follows. (**A**) Relative *Irf2bp2* mRNA levels in iWAT and eWAT. (**B**) Western blot analysis of IRF2BP2 and GAPDH (loading control) protein levels in iWAT and eWAT. Protein quantification was performed with imageJ. *P < 0.05 versus iWAT Ctrl; #*P* < 0.05 versus eWAT Ctrl. (**C**) Body weights (*n* = 9). (**D** and **E**) Weights of: (D) BAT and (E) iWAT and eWAT (*n* = 9). (**F**) H&E staining of iWAT (scale bar, 50 um). (**G**) Quantification of adipocyte size in iWAT (*n* = 7). (**H**) Circulating NEFA and glycerol levels under ad libitum fed conditions (*n* = 8). (**I**) Relative *Lipe* mRNA levels in iWAT and eWAT (*n* = 7). (**J**) Relative mRNA levels of *Pnpla2* and *Mgll* in iWAT and eWAT (*n* = 7). (**K**) Relative mRNA levels of adipocyte genes *Adipoq*, *Fasn*, *Scd*, and *Acly* in iWAT (*n* = 7). (**L**) Relative mRNA levels of inflammatory genes *Adgre1* (*F4/80*), *Il1b*, *Il6*, and *Ccl2* (*Mcp1*) in iWAT (*n* = 7). (**M** and **N**) Flow cytometry analysis of F4/80^+^; CD11c^+^ (CD45^+^) cells in stromal vascular cells from iWAT (M) and eWAT (N) (*n* = 3). In (A), (B), (E), (H), (I), (J), (L), and (M), unpaired two-tailed Student’s *t* tests were applied. Two-way ANOVA followed by Sidak’s test was applied for (G). *P < 0.05.

We next sought to determine the in vivo role of IRF2BP2 in adipocytes. To do this, we generated adipocyte-specific *Irf2bp2* KO (AKO) mice by interbreeding *Irf2bp2^flox^* and *Adiponectin* (*Adipoq*)-*Cre* mice. *Irf2bp2* mRNA and protein levels were significantly reduced in inguinal white adipose tissue (iWAT) and epididymal WAT (eWAT) of 12-week-old AKO compared to control mice (Fig. 3, A and B). Notably, *Irf2bp2* mRNA and protein levels were significantly higher in iWAT compared to eWAT (Fig. 3, A and B). AKO and control mice had similar body weights at 12 weeks of age (Fig. 3C). iWAT weight was reduced by ~25% in AKO mice compared to control mice, with no significant differences in either eWAT or brown adipose tissue (BAT) weights (Fig. 3, D and E). Hematoxylin and eosin (H&E) staining of iWAT sections showed that adipocyte size was reduced in iWAT of AKO compared to control mice, likely accounting for the reduced depot weight (Fig. 3, F and G). Circulating FFAs and glycerol were significantly elevated in AKO mice compared to control mice (Fig. 3H). Consistent with the in vitro adipocyte studies, *Lipe* expression was elevated in iWAT (2-fold increase) and eWAT (1.5-fold increase) of AKO compared to control mice (Fig. 3I). iWAT from AKO mice also expressed slightly higher levels of *Mgll* (Fig. 3J). *Irf2bp2* deficiency did not affect the expression of *Adipoq* or the lipogenic genes *Fasn*, *Scd*, and *Acly* (Fig. 3K). Circulating NEFA levels were also elevated in AKO compared to control mice housed at 30°C and exempt from thermal stress (fig. S3A). Treatment of mice with the β-adrenergic agonist norepinephrine (NE) induced circulating FFAs to higher levels of in AKO compared to control mice (fig. S3B). Fasting for 16 hours increased circulating NEFA levels and reduced WAT weights to similar levels in control and AKO mice (fig. S3, C and D).

Elevations in adipose tissue lipolysis and FFAs can be associated with macrophage recruitment, inflammation, and metabolic dysfunction (*36*, *37*). Consistent with this, proinflammatory genes, including the macrophage marker *Adgre1* (i.e., *F4/80*), inflammatory cytokines *Il1b* and *Il6*, and chemokine *Ccl2*, were upregulated in the iWAT of AKO versus control mice (Fig. 3L). Furthermore, flow cytometry analysis revealed a ~2.5-fold increase in the proportion of proinflammatory F4/80^+^; CD11c^+^ cells in the stromal-vascular fraction of iWAT from AKO versus control mice (Fig. 3M). The proportion of F4/80^+^; CD11c^+^ cells was also increased, but to a lesser extent, in the eWAT of AKO mice (Fig. 3N). In addition, the circulating levels of inflammatory cytokines, interleukin-6 (IL-6), IL-1β, and monocyte chemoattractant protein-1 (MCP-1) trended to be higher in *Irf2bp2* AKO compared to control mice (fig. S3, E to G). We did not observe a difference in circulating adiponectin levels (fig. S3H). Hepatic triglyceride levels were increased in AKO mice, suggesting that adipose tissue–derived fatty acids accumulated in liver (fig. S3I). Together, these data indicate that IRF2BP2 represses adipocyte *Lipe* expression and suppresses lipolysis under basal conditions in mice.

### Adipocyte IRF2BP2 deficiency causes glucose intolerance in mice

**Fig. 4.**
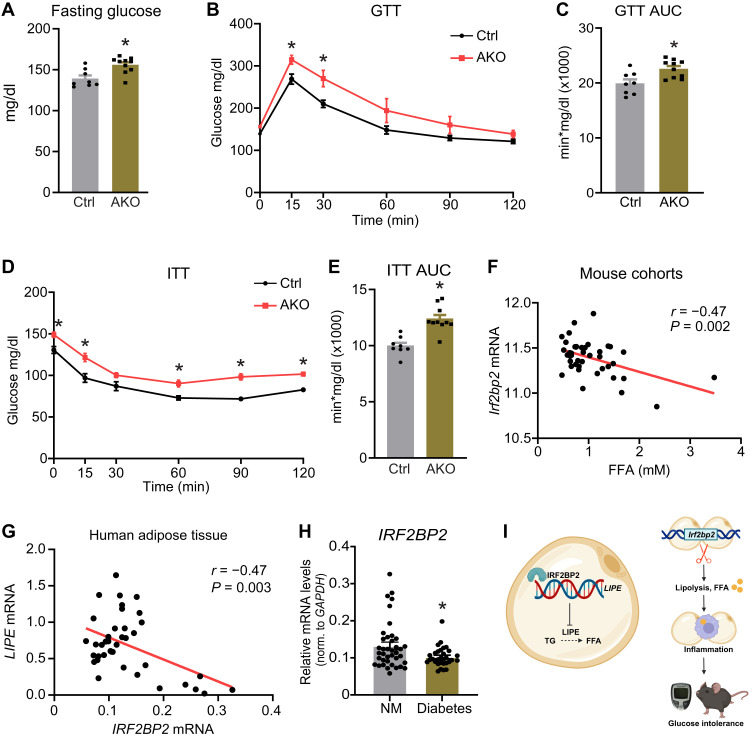
Adipocyte *Irf2bp2* deficiency causes glucose intolerance. (**A** to **E**) Analysis of metabolic parameters in 12- to 14-week-old control (Ctrl) and AKO mice (*n* = 8 to 10). (A) Fasting glucose levels. (B) Intraperitoneal GTT. (C) GTT AUC values from (B). (D) Intraperitoneal ITT. (E) ITT AUC values from (D). (**F**) Correlation between *Irf2bp2* mRNA levels in subcutaneous adipose tissue and serum FFA levels in 42 mouse strains (data extracted from GeneNetwork database, EPFL LISP3 Cohort) (*38*). (**G**) Correlation between *IRF2BP2* and *LIPE* mRNA levels in subcutaneous adipose tissue from healthy, nondiabetic female individuals (*n* = 39). (**H**) *IRF2BP2* mRNA levels in subcutaneous adipose tissue from diabetic (*n* = 31) and nondiabetic individuals (*n* = 39). (**I**) Working model: (Left) IRF2BP2 transcriptionally represses *LIPE* expression in adipocytes to decrease the rate of lipolysis. (Right) Loss of IRF2BP2 (or its down-regulation in diabetes) increases lipolysis, leading to inflammation and impaired metabolic homeostasis. In (A), (C), (E), and (H), unpaired two-sided *t* tests were used. Two-way ANOVA followed by Sidak’s test was used in (B) and (D). Person correlational analysis was performed in (F) and (G). *P < 0.05.

We further tested whether the action of IRF2BP2 in adipocytes regulates systemic glucose metabolism and insulin sensitivity. We found that fasting glucose levels were significantly elevated in AKO mice compared to control mice at 12 weeks of age (Fig. 4A). During a glucose tolerance test (GTT), *Irf2bp2* AKO mice exhibited higher blood glucose levels at each time point, with significant differences at 15 and 30 min (Fig. 4B). The area under the curve (AUC) for GTT was higher in *Irf2bp2* KO mice (Fig. 4C). AKO mice also displayed reduced glucose-lowering effects of insulin during an insulin tolerance test (ITT), with elevated blood glucose levels at 0, 15, 60, 90, and 120 min (Fig. 4, D and E). The data suggest that IRF2BP2-mediated suppression of basal lipolysis is required to preserve systemic insulin sensitivity and glucose homeostasis.

### IRF2BP2 expression levels correlate with markers of lipolysis in mice and humans

We next assessed whether IRF2BP2 expression levels correlate with markers of lipolysis in mouse and human cohorts. In the GeneNetwork cohort that analyzed 42 different normal mouse strains (*38*), *Irf2bp2* mRNA was significantly negatively correlated (*r* = −0.47) with circulating FFA levels (Fig. 4F). To explore associations between IRF2BP2 and lipolysis in humans, we collected subcutaneous adipose tissue samples from nondiabetic (*n* = 39) and diabetic individuals (*n* = 31) for expression analysis. In the nondiabetic human cohort (*n* = 39), *IRF2BP2* mRNA levels were negatively correlated (*r* = −0.47) with *LIPE* mRNA levels in subcutaneous adipose tissue (Fig. 4G). *IRF2BP2* mRNA levels were slightly (~20%) but significantly decreased in adipose tissue from patients with diabetes, compared to nondiabetic individuals (Fig. 4H). Together, these studies strongly support a conserved role of IRF2BP2 in restraining *LIPE* expression and lipolysis in mice and humans.

## DISCUSSION

Dysregulated adipocyte lipolysis drives the development of insulin resistance and cardiometabolic disease. In this study, we identified a role for the transcriptional cofactor IRF2BP2 as a repressor of adipocyte lipolysis. IRF2BP2 levels negatively correlate with lipolysis in mouse and human adipose tissue. IRF2BP2 gain-of-function and loss-of-function analyses in human adipocytes showed that IRF2BP2 is necessary and sufficient to suppress lipolysis. Last, adipocyte-specific deletion of *Irf2bp2* in mice elevated NEFA levels, associated with increased adipose tissue inflammation and systemic insulin resistance.

Our results suggest that IRF2BP2 mainly restrains basal adipocyte lipolysis, without influencing the lipolytic response to catecholamines. The pan β-adrenergic agonist isoproterenol activated lipolysis to similar extents in IRF2BP2-expressing or IRF2BP2-deficient adipocytes relative to their respective control adipocytes. Moreover, control and *Irf2bp2* KO mice exhibited comparably high levels of circulating NEFA levels following a 16-hour fast. The rate of basal (or resting) adipocyte lipolysis increases in obesity and correlates with cardiometabolic complications (*39*, *40*). Additional studies show that elevated lipolysis correlates with the early phase of impaired glucose tolerance in humans (*41*, *42*). Mechanistically, excess FFAs released from adipocytes trigger proinflammatory cytokine production (i.e., IL-6, IL-1β, and tumor necrosis factor–α) and provoke tissue inflammation (*43*, *44*). In line with this, adipocyte *Irf2bp2* deficiency led to adipose tissue inflammation and systemic insulin resistance in the absence of high-fat diet or weight gain. These results suggest that chronically high levels of basal lipolysis can trigger inflammation and insulin resistance.

Our results further suggest that IRF2BP2 controls lipolysis via regulating the transcription of *LIPE*. Previous studies revealed that upstream stimulatory factor and PPARγ bind to the *LIPE* promoter and modulate its transcription (*25*, *26*). Our ChIP-seq analysis indicate that IRF2BP2 binds to an upstream region in the human *LIPE* gene, representing a potential enhancer. Motif analysis of IRF2BP2 binding regions in adipocytes suggest that IRF2BP2 interacts with one or more member(s) of the AP1 family of transcription factors [e.g., activating transcription factor 2 (ATF2), JUN, FOS proteins] to regulate *LIPE* enhancer activity. Notably, IRF2BP2 was recently shown to interact and repress the function of the AP1 heterodimer ATF7/JDP2 in leukemia cells (*45*). Additional studies using JNK (upstream kinase of JUN) and p38 (downstream effector of ATF2) inhibitors (*46*, *47*) suggest that adipocyte lipolysis may be regulated by ATF2 and JUN, although the involved mechanisms remain unclear. Further studies are required to determine whether IRF2BP2 interacts with AP1 transcription factors to control LIPE expression and lipolysis.

Loss of *Irf2bp2* had a more pronounced effect in the subcutaneous iWAT as compared to the visceral eWAT depot. This may relate to the significantly higher expression levels of IRF2BP2 in iWAT. It is well established that iWAT and eWAT exhibit distinct responses to metabolic and inflammatory signals (*48*). eWAT undergoes higher rates of lipolysis (*48*) and harbors more proinflammatory macrophages (*49*). Loss of IRF2BP2 may be more consequential in iWAT because of the lower levels of lipolytic stimuli. Future studies are warranted to explore the differential regulation and roles of IRF2BP2 in different fat depots.

Reducing fatty acid levels can ameliorate insulin action and improve cardiometabolic health, underscoring the therapeutic potential of targeting this pathway (*3*, *5*, *50*–*52*). For example, treatment of mice with the ATGL inhibitor, atglistatin, lowers circulating fatty acids, decreases adipose inflammation, reduces liver fat, and ameliorates glucose homeostasis (*51*). Similar findings are observed in *Mgll* mutant mice, in *Lipe* haploinsufficient mice, and in mice treated with an HSL inhibitor (*3*, *52*). Thus, increasing the function of IRF2BP2 in adipocytes may provide a selective strategy to blunt basal lipolysis and reduce chronic inflammation.

## MATERIALS AND METHODS

### Mice

The mice used in our study were housed and cared for by the University of Pennsylvania University Laboratory Animal Resources in accordance with the guidelines set forth by the University of Pennsylvania Institutional Animal Care and Use Committee (IACUC). All animal procedures were performed following the guidance of the IACUC under approved protocol no. 805649. Mice were maintained at room temperature on a normal chow diet and subjected to a 12-hour light/dark cycle unless specified otherwise. *Adipoq-Cre* mice were obtained from the Jackson Laboratory [strain name: B6;FVB-Tg(*Adipoq-cre*)1dEvdr/J, RRID:IMSR_JAX:010803] (*53*). The *Irf2bp2* loxP/loxP strain was provided by A. Stewart (*28*). Mice were euthanized by CO_2_ asphyxiation, followed by cervical dislocation. Blood (fed condition) was collected by cardiac puncture and subjected to centrifuge for plasma NEFAs (HR series NEFA, WAKO) and glycerol (Randox) determination. Circulating adiponectin levels were evaluated by an enzyme-linked immunosorbent assay kit (Proteintech). Fat tissues were immersed in 4% formaldehyde for histology analysis or immediately cryopreserved for protein and mRNA analysis. For the thermoneutral studies, mice were housed at 30°C for 7 days. For NE stimulation studies, mice received an intraperitoneal injection of NE at a dose of 0.8 mg/kg, and blood was collected 60 min after injection for NEFA measurement.

### Mouse metabolic phenotyping

GTT and ITTs were conducted in male mice aged 12 to 14 weeks. For GTTs, a 6-hour fasting period preceded the intraperitoneal administration of 1.5 g of glucose per kilogram, delivered as a 20% d-glucose solution dissolved in sterile water. For ITTs, mice were fasted for 4 hours, followed by a bolus intraperitoneal injection of insulin (0.65 U/kg; Humulin, Novo Nordisk). Blood glucose levels were monitored from the tail-tip using an automatic glucometer (Contour Next, Bayer) at baseline and at 15, 30, 60, 90, and 120 min after injection.

### Flow cytometry analyses

Stromal vascular cells were isolated from adipose tissue depots for analyses. Dissected and minced adipose tissue was washed in phosphate-buffered saline (PBS) followed by digestion in Dulbecco’s modified Eagle’s medium (DMEM)/F12 medium (Gibco Invitrogen) containing dispase (2.4 U/ml), type I collagenase (1.5 U/ml; Gibco Invitrogen), and 1% bovine serum albumin (BSA) at 37°C with constant shaking for 30 min. The digestion was quenched with DMEM/F12 medium supplemented with 10% fetal bovine serum (FBS). The digested cell suspension was filtered through a 100-μm mesh filter (BD Biosciences) and centrifuged at 400*g* for 4 min at 4°C. Red blood cells were lysed by incubating cell suspensions in a hemolysis buffer for 4 min. Cells were suspended in FBS-containing medium and further filtered using a 70- and a 40-μm mesh, followed by centrifugation (400*g*, 4 min). Cell pellets were resuspended in fluorescence-activated cell sorting (FACS) buffer (0.5% BSA and 2 mM EDTA in PBS) and incubated with fluorophore-conjugated antibodies for 45 min at 4°C in the dark. The following antibodies were used: CD45–allophycocyanin (APC)/Cy7 (103116, BioLegend), F4/80-APC (123116, BioLegend), and CD11c-phycoerythrin (12-0114-82, eBioscience). 4′,6-Diamidino-2-phenylindole (DAPI) was used for viability staining. After three washes with FACS buffer, stained cells were suspended in 200 μl of FACS buffer for analysis. The CytoFlex Flow Cytometer (Beckman) was used to acquire 40,000 events for each sample. Unstained and single stain controls were used for setting compensation and gates. Debris, cell aggregation, and dead cells (DAPI^+^) were excluded. Macrophages were identified as CD45^+^;F4/80^+^, and M1 macrophages were identified as CD45^+^;F4/80^+^;CD11c^+^. Data were analyzed using FlowJo v10 (FlowJo).

### Cytokine multiplex array assay

Mouse plasma samples were diluted twofold with PBS and subjected to cytokine multiplexing analysis using the Luminex 200 system (Luminex, Austin, TX, USA) by Eve Technologies Corp. We used the mouse Focused 10-Plex Discovery Assay (MilliporeSigma) according to the manufacturer’s protocol.

### Hepatic triglyceride measurement

Liver samples weighing 50 to 100 mg were homogenized using a tissue lyser (25 Hz, 5 min) in a lysis buffer composed of 140 mM NaCl, 2.5% Triton X-100, 0.2% sodium deoxycholate, and 50 mM tris (pH 7.4). Triglyceride (TG) levels were quantified using the Stanbio Triglyceride LiquiColor kit. Following the manufacturer’s instructions, 2 μl of the sample or standard was added to 200 μl of the TG enzymatic mix, incubated for 10 min, and the reactions were subsequently assayed in a plate reader at 500 nm.

### hAPCs and adipogenic differentiation

hAPCs were obtained from subcutaneous adipose tissue samples collected through lipoaspiration. hAPCs were differentiated into mature adipocytes using DMEM/F12 medium supplemented with 5% FBS and treated with a cocktail consisting of 2.5 × 10^−8^ M insulin, 10^−5^ M dexamethasone, 5 × 10^−4^ M 3-isobutyl-1-methylxanthine (IBMX), and 10^−6^ M rosiglitazone for 14 days. For ChIP and luciferase assays, hAPCs were treated with differentiation cocktail without rosiglitazone.

### Lentivirus transduction

hAPCs were seeded in 60- or 100-mm culture dishes and transduced with lentivirus using the lentivirus viraductin kit from Cell Biolabs. Lentivirus used in the experiments were produced by human embryonic kidney–239T cells (American Type Culture Collection). Lentivirus for *IRF2BP2* KO was produced using lentiCRISPR v2 (Addgene plasmid no. 52961) carrying guide RNAs (gRNAs) targeting *IRF2BP2*. The gRNAs were designed using CRISPOR (*54*) and IDT CRISPR web design tools and synthesized by IDT. IRF2BP2 OE lentivirus was produced using plenti backbone plasmid (Addgene plasmid no. 17448) containing the full-length coding sequence cloned from hAPCs. Plasmid sequences were confirmed by Sanger sequencing (Genewiz).

### qRT-PCR and RNA-seq analysis

Tissue RNA was harvested using Trizol (Invitrogen) according to the manufacturer’s protocol. Reverse transcription reactions were performed with High Capacity cDNA Reverse Transcription Kit (Thermo Fisher Scientific). qRT-PCR was performed using PowerUp SYBR Green Master Mix (Thermo Fisher Scientific) on a QuantStudio 5 instrument (Thermo Fisher Scientific). Primers used for qRT-PCR reactions were summarized in table S1. Relative gene expression levels were calculated by the ddCt method using the housekeeping gene *GADPH* (human) or *Gapdh* (mouse) as control.

For RNA-seq, extracted cellular total RNA samples were sequenced by NovaSeq 6000 PE150 (Novogene). The raw paired-end Fastq files were trimmed using the tool TrimGalore (version 0.6.5) with default parameters and paired option enabled. The trimmed files were then aligned to the GRCh38 (version 104) genome using kallisto (version 0.46.1) with the default parameters of the kallisto quant command for paired-end data (*55*). Kallisto output files were read into R (version 4.3.0) and differentially expressed genes were generated by EdgeR (version 4.0.2) (*56*). Volcano plots were generated using the package ggplot2 (version 3.4.4) where genes with a *P* value less than 0.05 were colored on the basis of expression. The enrichment analysis was performed with gprofiler2 (version 0.2.2) using genes with a false discovery rate less than 0.1, positive or negative logFC cutoff, and being ordered by significance. A gSCS (Set Counts and Sizes) correction method was applied to the enriched terms, organism was set to hsapiens, ordered query to TRUE, a cutoff of 0.001 for significance, and using the “Biological Processes” as the source. The top enriched pathways were plotted into bar graphs using the ggplot2 package where the terms were ordered on the basis of *P* value for merged datasets. Heatmaps were generated using packages ComplexHeatmap (version 2.16.0) and pheatmap (version 1.0.12). Rows were scaled to their relative scores (*z*-score) for both heatmaps. In fig. S2, heatmaps rows were clustered in ComplexHeatmap by their Euclidean distances with a ward.D2 clustering method and a k-means clustering of 4. The Venn diagram was generated using genes with a positive or negative fold change and a false discovery rate cutoff of 0.1 in which genes were compared between the different datasets to evaluate overlapping differentially expressed genes across conditions for the RNA-seq.

### Lipolysis and glucose uptake in adipocytes

Adipocytes were serum starved overnight in DMEM/F12 medium. The cells were then treated with 2% BSA, serum-free medium containing either PBS (control) or 10^−6^ M isoproterenol for 4 hours, and culture medium was collected for NEFA assessment (HR series NEFA, WAKO) and glycerol assessment (Randox) following the manufacturer’s instructions. The absorbance was measured in a colorimetric plate reader at a wavelength of 550 nm. NEFA and glycerol values were normalized to protein content, which was determined using Pierce BCA Protein assay. Glucose uptake was determined using a Promega Assay Kit (J1342). Briefly, overnight serum-starved adipocytes were washed with PBS, followed by treatment with 10^−8^ M insulin for 1 hour in glucose-free medium. Cells were then subjected to 2-deoxyglucose uptake, acid termination, neutralization, and luminescence measurement.

### Western blotting

Cells were lysed in radioimmunoprecipitation assay (RIPA) lysis buffer [NaCl 150 mM, NP-40 1%, sodium deoxycholate 0.5%, SDS 0.1%, and 50 mM tris (pH 7.4)] containing protease inhibitors (Roche), phosphatase inhibitors (Thermo Fisher Scientific), and 100 μM phenylmethylsulfonyl fluoride (Sigma-Aldrich). Adipose tissue depots were homogenized in RIPA buffer using the Qiagen TissueLyser (28 Hz, 4 min). Tissue homogenates were kept on ice for 30 min with intermittent vortex, followed by two to three rounds of centrifugation (15 min, 12,000*g*) to remove cell debris and lipids. Protein concentration was determined by bicinchoninic acid (BCA) assay. Lysates (30 μg) were separated on precast 4 to 12% bis-tris NuPage gels (Thermo Fisher Scientific) and transferred to polyvinylidene difluoride membranes. Proteins were detected using primary antibodies against IRF2BP2 (A303-190A, Bethyl Laboratories), phospho-LIPE (4126S, Cell Signaling Technology, Danvers, MA), LIPE (4107S, Cell Signaling Technology), and glyceraldehyde phosphate dehydrogenase (GAPDH) (MA5-15738, Thermo Fisher Scientific). Horseradish peroxidase–conjugated secondary antibodies i.e., anti-mouse (7076S, Cell Signaling Technology) and anti-rabbit (7074S, Cell Signaling Technology) were used, followed by SuperSignal West Pico PLUS ECL detection (Thermo Fisher Scientific). Blotting images were scanned using Amersham ImageQuant 800 (Cytiva). Bands were quantified using ImageJ.

### Dual-luciferase reporter assay

Luciferase reporter assay was performed as previously described (*57*). Immortalized hAPCs were seeded in 48-well plates. Cells were treated with the differentiation cocktail of insulin, dexamethasone, and IBMX for 24 hours, followed by transfection using Lipofectamine 3000 method (Thermo Fisher Scientific). For one well, 0.75 μl of lipofectamine 3000 and P3000 diluted with Opti-MEM medium (Thermo Fisher Scientific) were used. Firefly luciferase enhancer reporter vector (200 ng) was cotransfected with 200 ng of IRF2BP2 or empty pcDNA3.1 vector, with 5 ng of phosphoglycerate kinase (*Pgk*) promoter-driven *Renilla* luciferase as a normalization control for transfection efficiency. Cells were harvested 48 hours after transfection, and lysates were subjected to luminescence measurement. Luciferase assay was performed using dual-luciferase reporter assay (E1910, Promega) according to the manufacturer’s instructions.

### ChIP and CUT&RUN sequencing

ChIP was performed as described previously (*58*). Cells were treated with the differentiation cocktail of insulin, dexamethasone, and IBMX for 24 hours before chromatin collection. Sonicated input sample (10% of total chromatin) was saved and subjected to cross-link reversing and column purification. Sonicated chromatin samples were incubated overnight at 4°C with IRF2BP2 antibody in 1 ml of ChIP buffer [50 mM Hepes (pH 7.8), 140 mM NaCl, 1% Triton X-100, 0.1% Na-deoxycholate, and Complete protease inhibitor]. Immunoprecipitation was washed and then eluted by incubation at 65°C overnight in 50 μl of ChIP elution buffer [50 mM tris (pH 7.5), 10 mM EDTA, and 1% SDS] with 10 mM dithiothreitol. Beads were pelleted, and the supernatant was transferred to a new tube. DNA was subsequently isolated by columns (NucleoSpin, Takara). H3K27ac CUT&RUN experiment was similarly performed in hAPCs following manufacturer’s protocol (86652, Cell Signaling Technology). DNA bound by H3K27Ac (Active Motif) or immunoglobulin G (IgG) (Millipore) was collected. ChIP and CUT& RUN libraries were prepared and sent for next-generation sequencing (Novogene). Sequencing data were analyzed as previously reported (*57*). Briefly, reads were aligned using Bowtie2 (version 2.5.0), followed by MACS2 (version 2.2.8) peak calling and Homer (version 4.11) annotation and motif enrichment. For the ChIP-qPCR, cells were differentiated into mature adipocytes for 14 days following cocktail treatment. Adipocyte nuclei were isolated by douncing and subjected to ChIP assays using antibodies against IRF2BP2 and IgG as control. The eluted DNA was analyzed by qRT-PCR using primers specific for the *LIPE* enhancer region. Binding was quantified relative to the input DNA signal.

### Histology

Tissues were fixed in 4% paraformaldehyde overnight, washed in PBS, dehydrated in ethanol, paraffin embedded, and sectioned. Sections were stained with H&E. Images were captured on a Keyence inverted microscope. Adipocyte size was quantified with ImageJ software.

### IRF2BP2 correlational studies in mice and humans

*Irf2bp2* mRNA transcript expression and FFA levels in 42 mouse strains were determined using data extracted from GeneNetwork database (i.e., EPFL LISP3 Cohort) (*38*). We assessed the mRNA levels of *IRF2BP2*, *LIPE*, and *GAPDH* (normalization control) in subcutaneous adipose tissue samples from nondiabetic (*n* = 39) and diabetic (*n* = 31) female individuals that underwent bariatric surgery with an approved institutional review board protocol at Hospital of the University of Pennsylvania.

### Statistical analysis

Data analysis was performed with GraphPad Prism 10 software. No power calculations were performed before initiation of the study. All individual data points were plotted to assay normality. Data normality was assessed with Shapiro-Wilk test. For normally distributed data, two-sided *t* tests were performed where comparisons between two groups were being assayed. Nonparametric Mann-Whitney test was applied for data that were not normally distributed. Two-way and one-way analysis of variance (ANOVA) with pairwise comparisons were performed where comparisons between more than two groups were being assayed in cell or mouse studies. Correlational analysis was performed using GraphPad.
