# Supplementary material for: Transcriptional regulation of adipocyte lipolysis by IRF2BP2
Source: Sci Adv. 2025 Jan 3;11(1):eads5963. doi: 10.1126/sciadv.ads5963 (PMC11698119; doi:10.1126/sciadv.ads5963)
Supplement: Supplementary file 1 — Figs. S1 to S3 Tables S1 and S2 [file sciadv.ads5963_sm.pdf]

Science Advances
AAAS

Supplementary Materials for

Transcriptional regulation of adipocyte lipolysis by IRF2BP2

Yang Chen *et al.*

Corresponding author: Patrick Seale, sealep@pennmedicine.upenn.edu

Sci. Adv. **11**, eads5963 (2025)
DOI: 10.1126/sciadv.ads5963

This PDF file includes:

Figs. S1 to S3
Tables S1 and S2

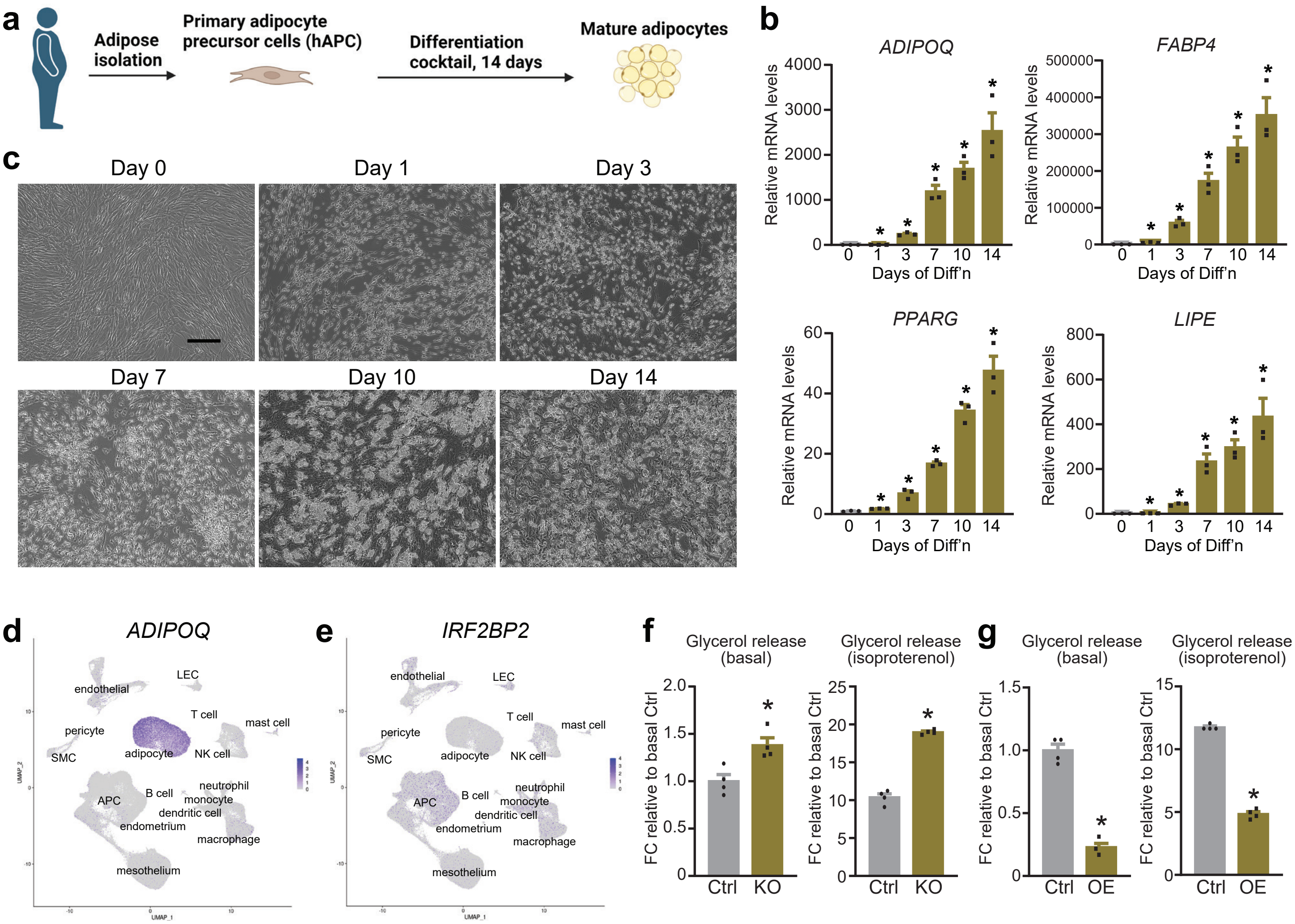

Fig. S1. IRF2BP2 expression during human adipocyte differentiation

a, Human adipocyte precursor cells (hAPC) isolated from subcutaneous adipose tissue were differentiated into mature adipocytes. n=3 per timepoint. **b**, Relative mRNA levels of adipocyte genes *ADIPOQ*, *FABP4*, *PPARG* and *LIPE* during differentiation. **c**, Phase contrast images of adipocyte cultures during differentiation (scale bar, 100 µm). **d-e**, UMAP of *ADIPOQ* and *IRF2BP2* expression in single nucleus RNA-seq dataset from human adipose tissue (*35*). **f-g**, glycerol release in IRF2BP2 knockout (KO) and overexpression (OE) adipocyte cultures under basal and isoproterenol (ISO) treated conditions. One-way ANOVA followed by Dunnett's test was used in b.

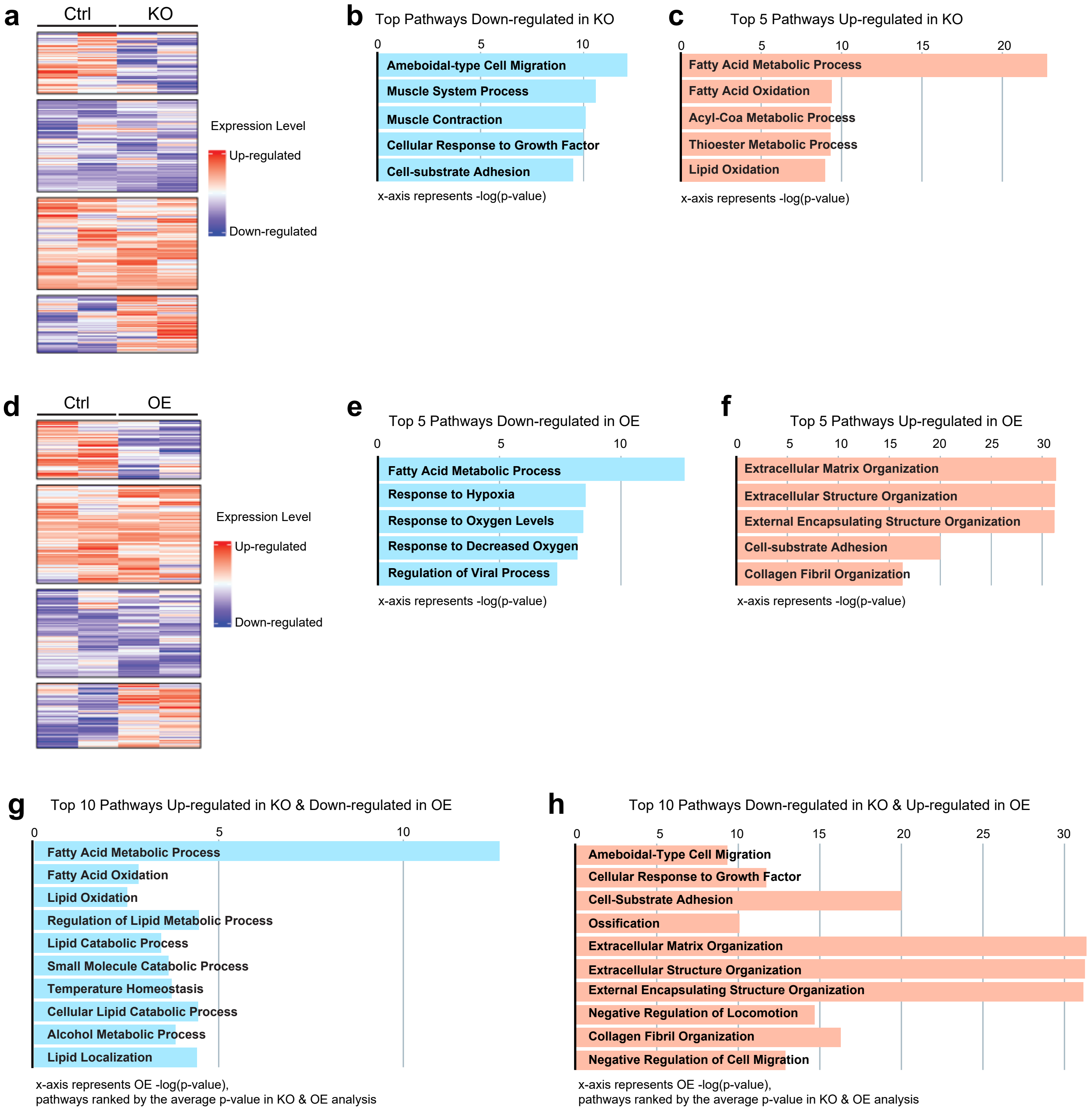

Fig. S2. Bulk RNA-seq heatmap and pathway analysis

a, Expression heatmap from RNA-seq analysis of Ctrl and KO adipocytes. **b,c**, Biological process (BP) enrichment in Ctrl vs. KO cells: (b) downregulated pathways; and (c) upregulated pathways. **d**, Expression heatmap from RNA-seq analysis of Ctrl and OE adipocytes. **e,f**, BP enrichment in Ctrl vs. OE cells: (e) downregulated pathways; and (f) upregulated pathways. **g,h**, BP pathways that are upregulated in KO and downregulated OE, in Ctrl vs. OE study: (g) downregulated pathways; (h) upregulated pathways.

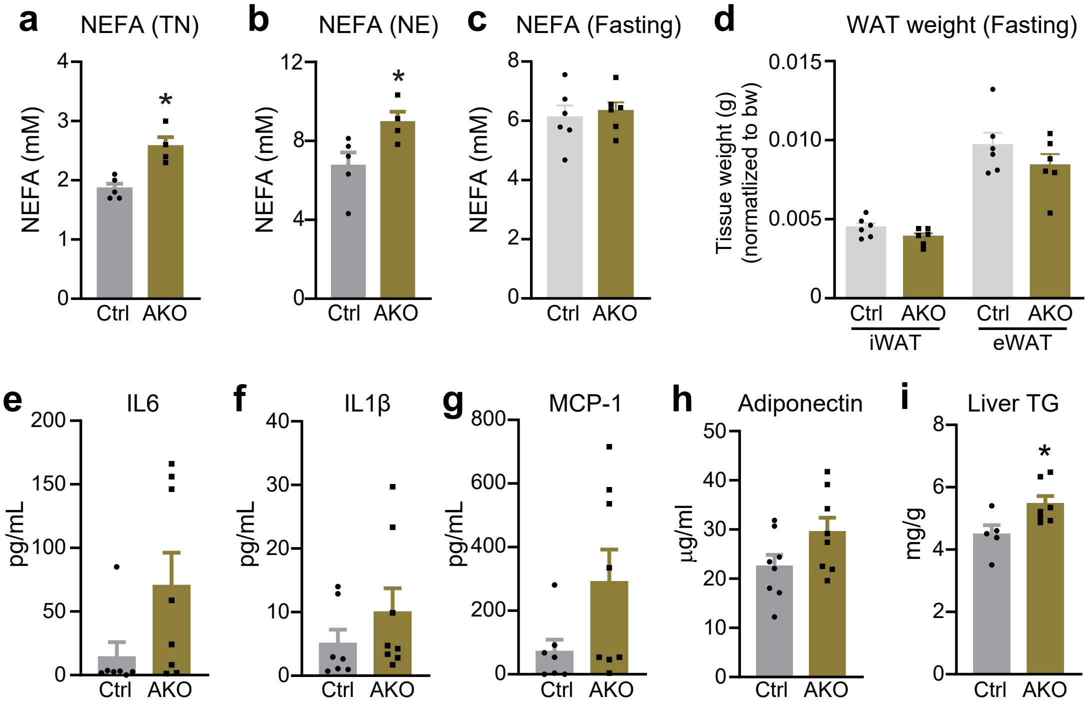

Fig. S3. Adipocyte IRF2BP2 regulates lipolysis, inflammation and metabolic parameters

a-c, Circulating NEFA levels in 12- to 14-week-old control and *Irf2bp2* AKO male mice under the following conditions: (a) housed at/near thermoneutrality (TN, 30C) for 7 days; (b) treated with 0.8 mg/kg norepinephrine 60 min before measurement; (c) following a 16-hour fasting period. n=4-7 mice of each genotype per condition. **d**, Adipose depot (iWAT, eWAT) weights in control and *Irf2bp2* AKO male mice (n=6, age 12-14 weeks) following a 16-hour fasting. **e-h**, Circulating levels of: (e) IL6, (f) IL1β, (g) MCP-1, and (h) Adiponectin in 12- to 14-week-old control and AKO male mice (n=7-8). **i**, Hepatic triglyceride levels 12- to 14-week-old control and AKO male mice (n=5-7).

Table S1. qRT-PCR and ChIP qPCR primer sequences

Gene	Forward	Reverse
GAPDH	TCCAAAATCAAGTGGGGCGA	AAATGAGCCCCAGCCTTCTC
IRF2BP2	CATGATCTGGGACTTCACCG	TCTCGATGACGAACTCGACG
PPARG	AACAGATCCAGTGGTTGCAGA	CATGAGGGAGTTGGAAGGCT
ADIPOQ	AACATGCCCATTCGCTTTACC	TAGGCAAAGTAGTACAGCCCA
FABP4	CATGTGCAGAAATGGGATGG	AACTTCAGTCCAGGTCAACG
LIPE	AGTGCTTCTTCGCCTACTGC	GCAGATTCGTTCCCCTGTTG
Gapdh	GGCATTGTGGAAGGGCTCAT	AGATCCACGACGGACACATT
Irf2bp2	AGTTCTGTTTCCCTTGCTCC	TCTTCACTTTCACATCTCCGG
Lipe	GGGTGATGAAGGACTCACCG	GATGGCAGGTGTGAACTGGA
Adgre1	CTCAGTCTGCACCAATATCCTG	CCACAGAGTTAGAGCAGTTGGAA
Il1b	GTGTCTTTCCCGTGGACCTT	AATGGGAACGTCACACACCA
Il6	AGAGACTTCCATCCAGTTGCC	CCGGACTTGTGAAGTAGGGAA
Ccl2	AGGTGTCCCAAAGAAGCTGT	AAGACCTTAGGGCAGATGCAG
Plnla2	TTCGCAATCTCTACCGCCTC	AGCAAAGGGTTGGGTTGGTT
Mgll	CGCGCAGTAGTCTGGCTCTA	ATTCTGTGGAGTTCGCCTGG
Adipoq	ATCTGGAGGTGGGAGACCAA	GGGCTATGGGTAGTTGCAGT
Fasn	GCTGCGGAAACTTCAGGAAA	GAGTTGAGCTGGGTTAGGGT
Scd	CGAGGGCTTCCACAACTACC	AACTCAGAAGCCCAAAGCTCA
Acly	ACCATCATTGGGCCAGCTAC	GACATGCCTCCTGAACGTGA
LIPE ChIP	TTGCACTCACATTCTTGGCC	GGAACTGTGCTGACCTAGGT

Table S2. Baseline characteristics of non-diabetic and diabetic subjects

	Age	Race, white %	Weight, kg	BMI	HbA1c, %	TC, mg/dL
Non-diabetic	40.1±1.1	48.7	125.7±2.9	45.8±1.4	5.5±0.05	180.6±4.8
Diabetic	43.6±1.2	32.2	135.4±4.5	49.8±1.1	6.4±0.16	182.7±6.7

Diabetes status is determined by patient records and medication history. BMI: body mass index. TC: total cholesterol.
